# Strategies of Recovery and Organic Recycling Used in Textile Waste Management

**DOI:** 10.3390/ijerph19105859

**Published:** 2022-05-11

**Authors:** Irena Wojnowska-Baryła, Katarzyna Bernat, Magdalena Zaborowska

**Affiliations:** Department of Environmental Biotechnology, University of Warmia and Mazury in Olsztyn, Sloneczna 45G, 10-709 Olsztyn, Poland; irka@uwm.edu.pl (I.W.-B.); magdalena.zaborowska@uwm.edu.pl (M.Z.)

**Keywords:** bio-based textile, biodegradability of textile waste, bioprocesses, bioethanol biorefining, biogas production, enzyme treatment

## Abstract

Post-consumer bio-based textile wastes are any type of garment or household article made from manufactured bio-based textiles that the owner no longer needs and decides to discard. According to the hierarchy of waste management, post-consumer textile waste should be organically recycled. However, there is still a problem with the implementation of selective collection of textile waste followed by sorting, which would prepare the waste for organic recycling. A technically achievable strategy for sorted textile waste materials consisting of only one type of fiber material, multi-material textiles are a problem for recycling purposes. Waste textiles are composed of different materials, including natural as well as synthetic non-cellulosic fibers, making bioprocessing difficult. Various strategies for recovery of valuable polymers or monomers from textile waste, including concentrated and dilute acid hydrolysis, ionic liquids as well as enzymatic hydrolysis, have been discussed. One possible process for fiber recycling is fiber recovery. Fiber reclamation is extraction of fibers from textile waste and their reuse. To ensure that organic recycling is effective and that the degradation products of textile waste do not limit the quality and quantity of organic recycling products, bio-based textile waste should be biodegradable and compostable. Although waste textiles comprising a synthetic polymers fractions are considered a threat to the environment. However, their biodegradable part has great potential for production of biological products (e.g., ethanol and biogas, enzyme synthesis). A bio-based textile waste management system should promote the development and application of novel recycling techniques, such as further development of biochemical recycling processes and the textile waste should be preceded by recovery of non-biodegradable polymers to avoid contaminating the bioproducts with nano and microplastics.

## 1. Introduction

In recent years, the textile industry has doubled production, while the time that clothing is worn before it is thrown away has fallen by around 40%. When it is thrown away, 73% of textile waste will be burned or landfilled, around 12% will be recycled, and less than 1% will be used to produce new clothing [1]. Polyester/cotton textiles are the most widely available types of textiles, in which their cotton fibers can be used as a feedstock for generating biofuels, e.g., ethanol, biogas. The cellulosic fraction of the world’s total fiber consumption is around 40%, the same proportion as the average cellulose content of lignocellulosic materials. Waste textiles are composed of different materials, including natural as well as synthetic non-cellulosic fibers, making bioprocessing difficult. In addition, around 90% of the global cellulosic fiber consumption is cotton, a material with a recalcitrant structure that makes it difficult to obtain a high yield in an enzymatic hydrolysis and biogas, and other value-added products [2].

The market offers a wide range of natural fibers, which are divided into three main groups, according to their origin: plant (cellulose) fibers, animal (protein) fibers, and mineral fibers [3]. The term man-made fibers refers to the process of manufacture and not the chemical composition of the material. Cotton and viscose are both fibers consisting of cellulose; cotton is a crop fiber and therefore classified as a natural fiber, and viscose is a man-made fiber produced by the viscose process [4].

Organic waste recycling is the process of organic waste management where organic wastes are recycled or converted into useful matter by different recycling methods. Organic recycling may offer the material a new lifecycle. The biobased textile wastes have a high potential to serve as an alternative feedstock for production of biological products via bioconversion of the cellulosic part of the textiles, which remained after the bioconversion as a purified value-added product.

Although, organic recycling may offer the materials a new lifecycle, this is limited by the large variety of fibers and colors used in fabrics, which present challenges for the sorting processes and decrease the quality of the recycled materials [5]. For any kind of reuse of textiles, they have to be collected separately from residual waste and sorted. Therefore, to increase the use of recycled material, there is a need for an economically viable and effective way to recognize and sort textile materials.

## 2. Methods Used for Textile Waste Recovery and Preparation for Their Recycling

### 2.1. Sorting of Textile Materials

Manual sorting of textile waste based on the fiber material content listed on product labels is possible but slow and often unreliable, because the labels may have been removed, be worn out or have faulty information [6]. Manual sorting is used to separate textiles by color, dye color, fabric type, quality and style.

There are methods available to identify textile materials, such as Fourier-transform infra-red spectroscopy (FTIR). This method could potentially be used to determine the color and fiber content of a textiles. However, it has not yet been implemented under real operating conditions. FTIR is possibly best viewed as a useful augmentation to manual sorting, since it can refine some of the steps of sorting by fiber type and color, and hence add value to output streams.

In future, radio frequency identification (RFID) tags could be used. These tags can be thought of as “wireless USB memory sticks” that can carry data and be remotely read. Low-cost RFID tags that can survive multiple laundry cycles do not yet exist. Two dimensional (2D) barcode labels could also carry information about textiles to inform sorting processes [7,8].

Girfanova and Abdyrasulova [9] proposed a textile waste sorting unit, after textile waste has undergone disinfection and dusting stages, it is necessary to remove fasteners, buttons, non-textile elements of products by press. Then, wastes moving along conveyor pass through spectral lamp, which transmits delta signal to robot. Installation for cutting zipper from textile products is characterized by the presence of two parallel-arranged disk knives permanently mounted above the table, equipped with a conveyor belt for the supply of textile products under the knives, having a guide middle chute for fixing the fastener-zipper, and side chutes for the operation of the disk knives and a front pressing roller for the supply of the textile product, and a rear roller for its removal.

Another option is near-infrared spectrometry (NIRS). This technique does not require sample preparation and is widely used in industry for a variety of purposes. NIRS has been proven to be capable of differentiating between unblended cotton, wool and polyester [10]. However, this technique has limitations related to the fact that it only analyzes textile surfaces. The thicker the layer facing the NIR sensor, the more difficulty it has in recognizing the material. Multi-layered samples may hide other materials at their core or under their visible surface, causing false positives and diminishing yields. A technically achievable strategy for sorted textile waste materials consisting of only one type of fiber material, multi-material textiles are a problem for recycling purposes. These multi-material textiles should therefore be avoided and a ‘design for recycling’ approach should be established in the textile and apparel industry. The potential of hyperspectral NIR cameras in reliably predicting the polyester content of natural and man-made cellulose and polyester blends is tested for recycling waste textiles [11].

### 2.2. Pretreatment Methods of Textile Waste

Various strategies for recovery of valuable polymers or monomers from cotton-based waste textiles, including concentrated and dilute acid hydrolysis as well as enzymatic hydrolysis, have been examined. For example, Shen et al. [12] developed a process for sugar and polyester recovery from cotton-based textile wastes via H_3_PO_4_ treatment. The maximal sugar recovery yield of 79.2% was obtained by treatment with 85% phosphoric acid for 7 h at 50 °C.

Many proposed solutions involve NaOH, which is a simple chemical that can swell cellulose at certain concentrations, and even dissolve it at high concentrations. Dissolution takes place when sodium hydrates penetrate the amorphous area of cellulose and destroy alline regions. A NaOH solution with a concentration of just 8–10% is sufficient for maximizing the solubility of cellulose that has a low to moderate degree of polymerization [13]. At cold temperatures, a combination of NaOH and urea can dissolve cellulose better than NaOH alone. NaOH destroys inter-and intra-hydrogen bonds between cellulose molecules, and urea hydrates function as hydrogen donors, and in some cases, as hydrogen receptors between solvent molecules, thus preventing the reassociation of cellulose molecules, and increasing the rate of molecular dissolution of cellulose [14,15]. However, this reaction may be inefficient because NaOH can react with the functional groups of cellulose instead of breaking down the bonds between cellulose molecules. With enzymatic pretreatment, it is possible to achieve a high degree of cellulose dissolution during treatment with a NaOH/urea solution. For example, Wang et al. [16] found that enzymatic pretreatment increased the solubility of cellulose in a cold NaOH/urea solution from 30% to 65%, which was mainly attributed to the reduction in the molecular weight of cellulose caused by the enzymatic treatment. 

Cotton cellulose has also been successfully degraded in an aqueous solution of NaClO under UVA light irradiation. Hydrolysis seemed to occur mostly on b(1 → 4)-glycosidic bonds in the cellulose polymers, and cleavage seemed to start from the ends of the polymer. From the photohydrolysis process, glucose was obtained, which can be used as resource for bioethanol [17].

The majority of textiles typically contain cellulose and a non-biodegradable polyester. When recycling these textiles, polyester recovery can be improved by alkaline pretreatment. For example, Gholamzad et al. [18] pretreated a textile containing cellulose and a non-biodegradable polyester with various alkaline solutions (NaOH (12 wt%), NaOH/urea (7/12 wt%), NaOH/thiourea (9.5/4.5 wt%) and NaOH/urea/thiourea (8/8/6.4 wt%)) at −20, 0, 23, and 100 °C for 1 h. These alkaline pretreatments, followed by hydrolysis, allowed 98% of the polyester to be recovered without significantly changing its properties.

For separating the cellulosic part of waste textiles, i.e., cotton and viscose, from other non-cellulosic fibers, the N-methylmorpholine N-oxide (NMMO) monohydrate process can be used. There are a number of advantages to this process for separating cellulose from waste textiles before it is bioconverted. Furthermore, the process can significantly increase the initial rate of enzymatic hydrolysis by 8–14-fold and the initial rate of biogas production by more than 15-fold [19]. Ionic liquids (ILs) are efficient direct solvents for cellulose materials and can be utilized for the chemical reprocessing of cotton. Recent studies on the application of ILs in cellulose chemistry, which include the production of regenerated and modified cellulose fibers, showed that they have various benefits, such as a low vapor pressure, better mechanical properties than those of commercial viscose fibers, and homogenous derivatization of cellulose that can be executed in one step. Preliminary investigations by Asaadi et al. [20] also indicated that ILs can selectively dissolve cellulose from a blend of cotton with polyester or nylon.

The many combinations of cations and anions that can be found in ILs give them a remarkably wide range of solvation that covers a variety of organic and inorganic materials. Due to their interesting properties, these liquids have been explored as promising solvents for the dissolution and fractionation of wood and cellulose, pulping, extraction of nanocellulose, and for processing all-wood and all-cellulose composites [21]. Imidazolium-based room-temperature ionic liquids (RTILs) have advantages over other pretreatment reagents, since they are nonvolatile, have low viscosity, and can be recovered and reused. Imidazolium-based chloride RTILs have been studied in the context of cellulose dissolution [22] and pretreatment prior to enzymatic hydrolysis [23] or acid hydrolysis [24,25]. [BMIM]Cl has been utilized most often, but it is a corrosive, toxic and extremely hygroscopic solid [22]. 1-Allyl-3-methylimidazolium chloride ([AMIM]Cl) is less toxic, less viscous and more readily synthesized, solubilizes cellulose better than [BMIM]Cl [26]. [AMIM]Cl has been used to pretreat microcrystalline cellulose before enzymatic hydrolysis [25], but not to treat cellulosic materials with a high crystallinity index and degree of polymerization, such as cotton-based textiles. According to Hong et al. [27], treatment of cotton cloth with [AMIM]Cl produced a substrate for the production of cellulose by *Gluconacetobacter xylinus*, provided that traces of the IL were removed after cellulose regeneration. For practical application of the IL-pretreatment technology, the cost of the ILs has to become lower, and recovery and reuse have to be improved.

The ionic liquid 1,5-diazabicyclo[4.3.0]non-5-ene acetate was identified as an excellent cellulose solvent that allows rapid dissolution at moderate temperatures and subsequent shaping into continuous filaments. The highly oriented cellulose fibers obtained upon coagulation in cold water exhibited superior tenacity, exceeding that of commercial viscose and NMMO-based Lyocell (Tencel^®^) fibers. The loncell yarn showed very good behavior during the knitting and weaving processes, reflecting the quality of the produced yarn [28].

Dissolution in a mixture of NMMO, [BMIM]Cl, and 85% phosphoric acid has been shown to be a very effective pretreatment for saccharification of dyed and blended waste fabric. The dissolution pretreatment significantly reduces the size of cellulosic fabrics, exposes the covered digestible native cellulosic fibers to cellulase, and reduces the crystallinity of the cellulose fibers. Saccharification conversion yields greater than 90% were achieved after this dissolution pretreatment [29].

The advantages of using ionic liquids as cellulose solvents instead of other methods include the relatively low processing temperatures, their recyclability, their thermal stability, their ability to tolerate low-quality raw material and their designability [30]. Thus, the potential of ionic liquids to enable textile recycling solutions is promising. For cellulose dissolution, a class of low-melting-point salt ILs has been used. However, despite their fascinating performance, ionic liquids are not harmless and green solvents. Many side reactions have been reported, including acetylation, hydrolysis, and thermal and chemical degradation, which significantly affect their recyclability. They also suffer from high viscosity and are still more expensive than commonly used organic solvents. However, the market for ILs continues to grow due to their inclusion in more applications and processes [21].

Wool is a complex natural fiber composed mainly of proteins (97%) and lipids (1%), and an ideal substrate for several classes of enzymes, such as proteases and lipases, which increase its rate of biodegradation [31]. Wool proteins in wool waste can be hydrolyzed by acids, bases, ionic liquids, reducing agents, thermal treatments, microorganisms or cell free proteases. In microbial hydrolytic processes, hydrolysis depends on microbial growth and enzyme secretion. Consequently, microbial hydrolysis takes longer than other methods, and microorganisms also consume part of the released amino acids for their growth. For preparation of wool hydrolysates, Zhang et al. [32] investigated the efficiency of wool degradation with the enzyme esperase (an endopeptidase with broad specificity) in combination with l-cysteine and urea. Hydrogen bonds, peptide bonds and disulfide bonds were cleaved with urea, esperase and l-cysteine, respectively. However, preliminary results showed that these substances were unable to degrade wool individually. Instead, a combination of all three components was most successful, with 99.5% weight loss. Holkar et al. [33] tested acoustic assisted alkaline hydrolysis of wool using a 20 kHz sonicator in combination with 24 h soaking of wool in 3% (*w*/*v*) KOH and 0.24% (*w*/*v*) NaOH, and 2% (*w*/*v*) wool loading, which were found to be the optimal conditions. The acoustic cavitation effectively broke disulfide bonds and inter- and intra-molecular hydrogen bonds.

### 2.3. Fiber, Polymer, Oligomer and Monomer Recovery from Textile Materials

One possible process for fiber recycling is fiber recovery. In this process, the textile is disintegrated into loose fibers, which can be spun into a yarn again. Fiber reclamation is extraction of fibers from textile waste and their reuse. Post-consumer waste is more difficult to recycle by this method than pre-consumer waste because of impurities left on the used products and their varying tear/wear condition.

A textile fiber is a unit of matter, either natural or manufactured, that forms the basic element of fabrics and other textile structures. The need for cellulose fibers is predicted to grow continuously due to their desirable technological properties. Cellulose is a recalcitrant polymer that is difficult to dissolve in common solvents. This has been attributed to the strong intramolecular hydrogen bonding present in cellulose [22]. To address this issue, the ionic liquid 1-butyl-3-methylimidazolium acetate ([Bmim]OAc) and dimethyl sulfoxide (DMSO) were used as solvents to dissolve waste cotton fabric, followed by regeneration of man-made cellulose fibers through a wet spinning process [34]. Similarly, cotton postconsumer textile wastes were fully solubilized in the cellulose-dissolving ionic liquid 1,5-diazabicyclo[4.3.0]non-5-enium acetate ([DBNH]OAc) for processing into continuous filaments. However, as a result of the heterogeneous raw materials that differed in molar mass distribution and degree of polymerization, it was necessary to adjust the degree of polymerization and ensure spinnability by pretreatment with acid hydrolysis, enzyme hydrolysis, or blending the waste cotton with birch prehydrolyzed kraft pulp. This allowed preparation of fibers with a tenacity (tensile strength) of up to 870 MPa, which exceeds that of native cotton and commercial man-made cellulosic fibers [20].

Cotton fibers and wood can be recycled with the Lyocell process, which is a cellulose regeneration process. In this process, cellulosic material is first dissolved in N-methylmorpholine-N-oxide (NMMO) and then pressed through a spinneret into water or an aqueous NMMO solution, where it coagulates and forms a new fiber [35].

To regenerate cotton fibers and separate them from other organic components (remaining from dyes) and polyester, a cleaner and sustainable chemical treatment using switchable hydrophilicity solvents (SHS) (as N, N-Dimethylcyclohexylamine (DMCHA) supplied by Sigma-12 Aldrich Corp., Seoul, Korea) was used to separate all layers of multilayer packaging (food and blister), including polyester layers [36]. In this approach, the polyester and organic component was extracted by dissolving it in a solvent and then changing the hydrophilicity of the solution by adding CO_2_.

A chemical technology has been developed by Ma et al. [34] for recovering cotton fibers and polyester from textile waste with a high recycling rate. In the first stage, textile dyes are leached from the fabric. In the second stage, the polyester is dissolved in green SHS, after which the cotton fibers are liberated from the fabric. Finally, the polyester is extracted from the solution, and the spent acid is treated with activated carbon to absorb the dyes.

Yousef et al. [37] tested a strategy for recovery of cotton from textile waste that consisted of three main steps: (i) as pretreatment, nitric acid was used to leach textile dyes from the original waste; (ii) organic materials, including polyester and the remaining organic parts of textile dyes, were dissolved using dimethyl sulfoxide (DMSO); (iii) for final purification of the recovered cotton, sodium hypochlorite and diluted hydrochloric acid were used for bleaching. The thermal stability of the recovered fibers was close to that of raw cotton, and they could be used as a new source of cotton after spinning by wet or melt spinning, which was only minimally limited by degradation.

Post-consumer waste is more difficult to recycle by alkali (LiOH or NaOH)/urea aqueous systems than pre-consumer waste because of impurities left on the used products and their varied tear/wear conditions. However, these systems have been used to solubilize and chemically recycle cotton waste by Liu et al. [38]. They hydrolyzed post-consumer cotton fabrics with H_2_SO_4_ and dissolved them in the above-mentioned solvents. Then, they successfully produced regenerated fibers via wet spinning. Good spinnability was observed with spinning solutions with a wide range of viscosities.

Polycotton, which is a mixed textile containing cotton and poly(ethylene terephthalate) (PET) polyester, is one of the most common materials in service textiles used in sheets and towels in hospitals and hotels. One of the difficulties in recycling cotton-based textiles is that they are commonly blended with different ratios of polyester, and the well-organized blending structure can obstruct enzymes from accessing the cotton. Blended cotton has higher degrees of crystallinity and polymerization than unblended cotton, due to the blended cotton’s higher molecular weight and the inter- and intra-molecular bonds that are present [39]. To hydrolyze the PET in polycotton, Palme [40] evaluated a straightforward lab-scale process. This process employed 5–15 wt% NaOH in water and temperatures ranging from 70 to 90 °C. In the process, PET was degraded to terephthalic acid (TPA) and ethylene glycol (EG). Three product streams were generated: cotton, TPA, and filtrate containing EG and process chemicals. With the addition of a phase transfer catalyst (benzyltributylammonium chloride (BTBAC)), PET hydrolysis at 90 °C in a 10% NaOH solution could be completed within 40 min. The yield of the cotton cellulose was high, up to 97%, depending on how long the samples were treated. The separation could be performed without the phase transfer catalyst, but this required longer treatment times, resulting in more cellulose degradation [40].

For recovering glucose and PET via bioconversion of textile wastes, Li et al. [41] developed a process that requires low enzyme input. The maximum glucose recovery (98.3%) was obtained with 20 FPU/g of cellulase and 10 U/g of β-glucosidase at 3% (*w*/*v*) substrate loading, a temperature of 50 °C and a pH of 5. The recovered PET from the textile waste was re-spun into new fibers by melt spinning.

To selectively degrade wool fibers while recovering synthetic fibers, Navone and Speight [42] proposed enzymatic treatment of wool/polyester fabric blends. A protease for keratinolytic activity and reducing agents were applied to fabric blends. The best results were obtained with a two-step enzymatic process in the presence of sodium thioglycolate. The polyester fibers were suitable for recycling into polyester yarn and then re-using in the manufacture of new garments or other products [43].

Stepwise enzymatic hydrolysis of cellulose/wool/polyester blends was shown to be effective by Quartinello et al. [44]. Textile waste was sequentially incubated with (1) protease to extract amino acids from wool components (95% efficiency) and (2) cellulases to recover glucose from cotton and rayon constituents (85% efficiency). Then, the amino acids and oligopeptides from wool degradation can be used to replace carbon and nitrogen sources. Similarly, the glucose from cotton hydrolysis can be converted into ethanol by fermentation with *Saccharomyces cerevisiae*.

Bio-based lignocellulosic (LC) materials have been used to prepare composites since historical times. For hybrid biodegradable composites, plant fibers were initially used mostly as short fibers, and later as woven or non-woven mats/fabrics. The latter were normally obtained using textile engineering techniques [45].

Old textiles are used to produce paper, particularly in conventional methods for making high quality paper. The paper making industry is an upcoming industry that has found that old fabrics are an excellent binding/connective material, e.g., old cotton textiles can serve to build the matrix of handmade paper. The recycled fibers are used to make paper, which is further used for creating tea bags, carry bags, envelopes and books.

PET is one of the most widely used synthetic polymers in the global textile industry. PET is a semicrystalline thermoplastic polymer which displays excellent tensile strength and chemical resistance, and high thermal stability. Two grades of PET dominate the global market: fiber-grade PET and bottle-grade PET [46]. The most common chemical-based PET hydrolysis processes involve alkaline hydrolysis using 4–20% NaOH/KOH solutions [47], phase transfer catalysts, or acidic hydrolysis with concentrated sulfuric acid or other mineral acids [48]. Unfortunately, classical chemical modification of synthetic polymers using strong alkaline or acid agents requires large amounts of energy and chemicals (binders, coupling agents, etc.), which are partially discharged to the environment. Although alkaline products render synthetic fibers more hydrophilic, they also cause deterioration of other properties, leading to irreversible yellowing and loss of resistance [49]. A chemo-enzymatic treatment has been developed to recover PET building blocks, namely terephthalic acid (TA) and ethylene glycol by Quartinello et al. [50]. According to those authors, chemical pretreatment under neutral conditions leads to depolymerization of the polyester-composed waste textiles, yielding about 85% TA. Then, enzymatic hydrolysis performed in a second reaction step leads to further hydrolysis of the remaining oligomers, yielding TA with a purity of 97%. Future studies should consider the chemo-enzymatic treatment of different PET-containing textile wastes as well as the synthesis of PET based on recovered TA.

Separation and recycling of waste nylon/cotton blended fabrics as raw materials for different products are attractive. A novel process for separating cellulose and nylon 6 from blended fabrics via an ionic liquid has been presented by Lv et al. [51]. Dissolution with [AMIM]Cl provides a simple and efficient process with a high rate of recovery of both regenerated cellulose films and nylon 6 fibers.

## 3. Biodegradability of Textile Waste

Biodegradable textile fibers can be defined as materials obtained from nature or by a synthetic route whose chemical bonds can be cleaved by enzymes. The suitability of such materials for a specific application will be dictated by their mechanical properties and their degradability. Most biodegradable textile materials, including composites, degrade within 2 weeks to 6 months [45].

The biodegradability of textiles is most likely influenced by their crystallinity, degree of orientation, degree of polymerization (DP), hydrophilicity/hydrophobicity, the condition of the soils where they are buried, and the species of microorganisms.

To biodegrade natural fabric samples (cotton, jute, linen, wool), two methods have been used: a standard method in which textile materials were directly degraded in soil, and an indirect method (non-standard method) in which the materials were sewn in bags and exposed to soil. The time required to biodegrade fibers containing cellulose can vary. Biodegradation of linen was fastest, which was connected with the structure of the linen fabric, whereas the degree of biodegradation of a linen/PET (poly(ethylene terephthalate) (PET)) blend material was lowest, which was also linked to the structure of the material and the fact that the linen was blended with PET, which showed no effects of degradation. Wool is rather resistant to the attack of microorganisms because of its molecular structure and its surface. These two factors make it quite difficult for the microorganisms to penetrate the structure of the wool and biodegrade it [52]. Rapid fabric biodegradation is facilitated by moist, warm aerobic soil conditions; under these conditions, the half-lives of rayon, cotton and Tencel^®^ were 22, 40, and 94 days, respectively [53].

The degradability of denim, silk and cotton material has been tested according to the ASTM D 5988-03 test, in a soil burial test and under sun exposure. In both the ASTM test and the soil burial test, silk was the most degradable textile and denim the least degradable. Under sun exposure, denim was the most degradable, and cotton the least degradable [54].

Cotton jersey fabrics with three finishing treatments (scoured and bleached, softener added and resin added) and a polyester jersey fabric were tested for biodegradability and compostability. The polyester fabric remained intact during the ASTM D 5988-03 test and the compost process. The cotton fabric with softener had an accelerated degradation rate, while the cotton fabric with resin had a relatively slow degradation rate. All cotton samples were significantly more degraded in the composting process than in the ASTM D 5988-03 test [55].

The cellulose fibers used in textiles do not have the same biodegradable behavior because of differences in their chemical and physical characteristics. Cotton, linen, rayon and acetate all contain celluloses but differ in terms of chemical composition, crystallinity, degree of polymerization and manufacturing process. The noncellulose content of fibrous materials differs in terms of its amount and composition. Park et al. [56] reported on associations between the properties of cellulose fibers and their biodegradability: moisture regain and biodegradability were most strongly associated.

It is well documented that cotton fibers, which are mainly composed of cellulose, are highly susceptible to microbial degradation. Microorganisms that cause the hydrolytic and oxidative degradation of cellulose belong to various genera (*Chaetomium* sp., *Fusarium* sp., *Myrothecium* sp., *Memnoniella* sp., *Stachybotrys* sp., *Verticillium* sp., *Alternaria* sp., *Trichoderma* sp., *Penicillium* sp., *Aspergillus* sp., *Cytohaga* sp., *Cellulomonas* sp., *Cellvibrio* sp., *Bacillus* sp., *Clostridium* sp. and *Sporocytophaga* sp.) [57]. Fungi and bacteria degrade cotton fabric in two different ways.

Fungi begin to attack fibers from the inside and work their way to the outer layer of fibers. In contrast to fungi, bacteria begin to attack fibers from the surface and work their way inwards. In the process of cellulose degradation, bacteria are less important than fungi because they need a higher percentage of moisture, which requires the fabric to be saturated throughout the whole process of degradation [58].

The type of dye affects the rate of biodegradation of cotton textiles. Under soil burial conditions, naturally colored cottons, and particularly green cottons, biodegrade more slowly than dyed or undyed conventional cottons because most synthetic dyes used to dye cotton are azo direct and reactive dyes. These dyes contain an azo bond, –N≡N–, which can be readily reduced or oxidized by aerobic or anaerobic bacteria. In contrast, natural colorants are more complex and thus more difficult for aerobic or anaerobic bacteria to reduce and/or oxidize [59].

Major end-use products of disposable nonwovens include wipes, absorbent hygiene products, medical products, filtration products, and protective garments. The use of raw materials derived from natural resources, such as cotton, rayon, and polylactic acid (PLA), has become of great interest to industries. Management of this type of waste requires determining the rate of biodegradation to enable the design of biological recycling methods [60]. For the biodegradation rates of raw cotton and rayon fabrics in a Captina silt-loam soil, a first-order kinetics model with respective half-life values of 12.6 and 7.6 days was fitted to the data. The half-life decreased as the temperature and/or moisture content of the soil increased. After 140 days of burial, PLA and PP were not significantly changed. PLA, derived from a natural source, was resistant to biodegradation [60].

In cellulose biodegradation, cellulose macromolecules are depolymerized, which is reflected by decreases in molecular weight and strength, an increase in solubility and a change in crystallinity.

When cellulose is modified, this influences the ability of microbes to degrade it. For example, modification of cotton fibers with a non-formaldehyde-containing finish based on imidazolidinone retarded biodegradation. This was due to formation of covalent bonds between the hydroxyl groups of the finish and strengthening of the cellulose molecules in the less ordered amorphous regions, which decreased fiber swelling. The modified cotton was more hydrophobic, and as a result, less wettable. Thus, the increase in macromolecular arrangement and the decrease in the amount of moisture in the finished fibers impaired the growth of microorganisms [61].

In addition to the cellulase enzymes that hydrolyze cellulose, which is a natural substance, there are also enzymes that can hydrolyze man-made synthetic fibers, such as polyester, and so-called biodegradable polymers, such as polylactic acid [62].

The wool textile industry produces a large amount of fibrous proteins, such as collagen, elastin and keratin, and in degradation of keratin-rich wastes from the wool textile industry, keratinolytic enzymes play an important role. Keratinases are the only group of proteases capable of degrading complex proteins over a wide range of pH values and temperatures [63]. Only a few bacteria, actinomycetes and fungi species are known to use keratin as source of carbon, nitrogen, sulfur and energy [64]. These microorganisms can be found in both aerobic and anaerobic environments and have been isolated in places ranging from hot springs to Antarctic soils [65]. The most intensively studied keratinolytic microorganisms belong to the bacterial genera Bacillus, Chryseobacterium, Serratia and Stenotrophomonas, as well as some actinomycetes (e.g., *Streptomyces* spp.) and some fungi (*Chrysosporium* spp., *Aspergillus* spp.). There are also some other enzymes that could be important for keratinous substrate degradation. It was recently found that specialized enzymes called lytic polysaccharide monooxygenases (LPMOs), which are important in cellulose and chitin degradation, could help break down keratin or keratinaceous matrix components. LPMO genes were also found in the dermatophytic and keratin-degrading fungi *Onygena corvina* [66]. Liu et al. [67] reported that γ-glutamyl transpeptidase and glyoxal/methylglyoxal reductase potentially play roles in keratinolysis by *Bacillus subtilis CH1*.

The ability to enzymatically degrade PET is thought to be limited to a few fungal species, and biodegradation is not yet a viable remediation or recycling strategy. Once identified, microorganisms with the enzymatic machinery needed to degrade PET could be useful for an environmental remediation strategy as well as a degradation and/or fermentation platform for biological recycling of PET waste products.

There are very few reports on the biological degradation of PET or its utilization to support microbial growth. The hydrolysis of PET fibers by two fungal hydrolases was investigated by Nimchua et al. [68]. The hydrolase from a newly isolated *Fusarium oxysporum* strain (LCH 1) released terephthalic acid from PET fibers more efficiently than the enzyme from *F. solani* f. sp. *pisi DSM 62420* under conditions of equal amounts of p-nitrophenyl butyrate-hydrolyzing activity. The enzyme from *F. oxysporum LCH 1* also increased the hydrophilicity of PET fabrics to a considerably greater extent than the hydrolase from *F. solani* f. sp. *pisi DSM 62420* [68]. The enzyme treatment clearly decreased the hydrophobicity of the PET fabric. Similarly, increases in water and moisture absorption resulting from treatment of PET fabrics were also observed by Fischer-Colbrie et al. [69] after treatment by enzymes from *Aspergillus* sp. *strain St 5* and by Alisch-Mark et al. [70] after treatment by enzymes from *Thermomonospora fusca*. Although aromatic polyesters have been considered to be very resistant to degradation by hydrolytic enzymes [71], there is accumulating evidence that esterases and cutinases from various fungi and bacteria can hydrolyze ester bonds in PET [68,72].

By screening natural microbial communities exposed to PET in the environment, Yoshida et al. [73] isolated a novel bacterium, *Ideonella sakaiensis* 201-F6, that is able to use PET as its major energy and carbon source. When grown on PET, this strain produces two enzymes capable of hydrolyzing PET and mono (2-hydroxyethyl) terephthalic acid, a reaction intermediate. Both enzymes are required to enzymatically convert PET efficiently into its two environmentally benign monomers, terephthalic acid and ethylene glycol.

Polyamide 6.6 (nylon) degradation has been investigated with a laccase-mediator system [74] and with proteases [69,75]. Nylon oligomers can also be biodegraded by microorganisms, such as Flavobacterium sp. and *Pseudomonas aruginosa* [76]. Additionally, polyamides have been depolymerized with oxidases from lignolytic fungi [77].

## 4. The Bioprocesses for Organic Recycling of Textile Waste

### 4.1. The Bioprocesses of Bioethanol Biorefining and Biogas Production from Textile Waste

Biodegradation of waste textiles, in contrast to biodegradation of lignocellulose, does not face problems caused by the presence of lignin and hemicellulose; instead, the major obstacle is the very high crystallinity of the cellulose in the cotton fibers. Although waste textiles comprising a synthetic polymer and cellulose fractions are considered a threat to the environment, their cellulosic part has great potential for biofuel production. Cellulose constitutes approximately 35–40% of textile waste, which could become a potential feedstock for production of biological products (e.g., ethanol and biogas) [12,19]. The cotton content in textile waste is considered an alternative source for producing renewable energy, and it has been investigated as a feedstock in the bioprocesses of bioethanol biorefining and biogas production.

#### 4.1.1. Ethanol Fermentation of Textile Waste

Pre-treatment is considered a crucial step in bioethanol production, and the search continues for the best methods of accomplishing this. Pre-treatment of cellulose feedstocks should increase their surface area and accessibility to enzymes by changing their porosity and decreasing their crystallinity. For this purpose, there have been tests of different chemical (alkali, organosolv), physico-chemical (hydrothermal, microwave-assisted acid pretreatment) and combined pretreatment methods (organosolv followed by hydrothermal treatment). Dimos et al. [78] found that the highest ethanol production of 32.3 g/L (corresponding to an ethanol yield of 47.6%) was achieved with sequential combination of organosolv and hydrothermal pretreatment as well as 6 h pre-hydrolysis when the highest glucose concentration was observed.

Alkaline pretreatments of textile waste can generally improve ethanol yields. Gholamzad et al. (2014) [18] pretreated polyester–cotton textile by different alkali solutions (details given in Section 2.2. Pretreatment methods of textile waste). Then, treated and untreated textiles waste was subjected to 72 h enzymatic hydrolysis at 45 °C and pH 4.8. The pretreatment conditions at which the highest hydrolysis yield was obtained was considered for the study of ethanol production through simultaneous saccharification (using S. cerevisiae) and fermentation (SSF). Authors indicated that all pretreatments improved the ethanol yields. With the use of NaOH/urea at −20 °C as a pretreatment, the highest ethanol yield of 70% was achieved. In comparison, the yield of untreated textiles was 36%.

Cotton waste from textile mills was processed with acid and alkali pretreatment to expose the sugars for enzymatic hydrolysis by the cellulase produced by *Fusarium* species. The acid pre-treated substrate improved enzyme activity and released more sugar than the alkali pre-treated substrates. The sugars were then fermented with *Saccharomyces cerevisiae* using simultaneous saccharification and fermentation. The amount of alcohol produced via batch fermentation was 11.8 mg/mL [79].

Viscose textile waste was a better source for ethanol synthesis than cotton textile waste. Morphological studies and measurements of enzymatic hydrolysis yields showed that alkali pretreatment of viscose waste fibers is not necessary, but that it should be performed with cotton waste fibers. More enzymatic hydrolysis took place and higher fermentation yields were produced with viscose fibers than with cotton ones (8.1 vs. 6.9 g/L), due to differences in the microcrystalline structures of the fibers [80].

Dilute sulfuric acid was used to pretreat cotton waste fibers, and the mixture was heated at a high temperature for 72 h to break the crystalline structure of the cellulose. *Saccharomyces cerevisiae* was used for fermentation, and about 14.5 mL of ethanol was recovered from the broth by distillation [81].

To recover cellulose from blended fiber waste textiles, N-methylmorpholine-N-oxide (NMMO) was used to separate and pretreat the cellulose. Next, the cellulose was hydrolyzed by cellulase enzymes, then fermented to ethanol. The process was tested with 50/50 polyester/cotton and 40/60 polyester/viscose-blended textiles. The polyesters were purified as fibers after the NMMO treatments, and up to 95% of the cellulose fibers were regenerated and collected. With 2-day enzymatic hydrolysis and 1-day fermentation of the regenerated cotton and viscose, the yields were 48 and 50 g ethanol/g regenerated cellulose, which were 85% and 89% of the theoretical yields, respectively [19].

Enzymatic hydrolysis of corona pre-treated mercerized and non-mercerized commercial cotton fabrics was used for ethanol fermentation. Both mercerization and corona pre-treatment increased the glucose concentration during enzymatic hydrolysis, which consequently increased the ethanol yield during fermentation. The volumetric productivity was much higher in the system with mercerized cotton fabric (three times higher than with the corona pre-treated mercerized cotton fabric). In these experiments, the mercerized cotton fabric was selected as the most efficient material for bioethanol production [82].

Ammonia was used to pretreat a mixture of waste cotton and cardboard that was then saccharified with in-house cellulases produced by *Trichoderma harzanium* ATCC 20846. The saccharification percentage was 45% and the percent yield of saccharification was 94.6%. Optimized fermentation with *Saccharomyces cerevisiae* strain RW143 produced a yield of 0.4 g ethanol/g glucose from enzymatically saccharified hydrolysate with a glucose load of 15% (*v*/*v*). From 1 kg of biomass mixture, the ethanol yield was 120 mL [83].

In the process of hydrolysis, cellulose is broken down to free sugar molecules by the addition of water, which is also called saccharification. Hydrolysis takes place after pretreatment, which breaks down the feedstocks into fermentable sugars for bioethanol production. Acidic and enzymatic hydrolysis are the two most common methods. Acidic hydrolysis is the oldest and most commonly used method [84]. As the name would imply, in enzymatic hydrolysis, enzymes hydrolyze the feedstocks to fermentable sugars.

In bioethanol production, three processes are commonly used: separate hydrolysis and fermentation (SHF), simultaneous saccharification and fermentation (SSF) and simultaneous saccharification and co-fermentation (SSCF). Both SSF and SSCF are preferred over SHF because the operation can be performed in one tank, resulting in lower costs, higher ethanol yields and shorter processing times [85].

Typically, Saccharomyces cerevisiae yeast is used for bioethanol production. Certain yeast strains such as *Pichia stipitis* (NRRL-Y-7124), *S. cerevisiae* (RL-11) and *Kluyveromyces fagilis* (Kf1) have been reported to be good for producing ethanol from different types of sugars [86].

#### 4.1.2. Anaerobic Digestion of Textile Materials

Hydrolysis is widely acknowledged as the rate-limiting step in anaerobic digestion of solid cellulose to biogas (methane), and pretreatment is generally thought to facilitate the process. Jeihanipour et al. [87] compared the solubilization rate of anaerobic digestion of cotton linter (high crystalline cellulose) with that of regenerated cellulose (amorphous cellulose) using pretreatment with NMMO. The maximum solubilization rates of treated cellulose were 842 and 517 mg COD/(g COD∙day) at initial cellulose concentrations of 5 and 30 g/L, respectively, while the solubilization rate of untreated cellulose never exceeded 417 mg COD/(g COD∙day). The difference between the two cellulose types was a direct result of the high rate of hydrolysis inhibiting the activity of microorganisms performing acetogenesis/methanogenesis, a drawback to the rest of the process. One possible solution for benefitting from the high solubilization rate of pretreated cellulose, along with an increased loading rate, is to use a two-stage anaerobic digestion process, where hydrolysis and acidogenesis of the cellulose can proceed in the first reactor, while the volatile fatty acids that are produced can subsequently be converted into biogas in the second reactor.

Biogas production from cotton/polyester and viscose/polyester with no pretreatment or milling revealed that gas production efficiency is strongly affected by the molecular structure of the textile [88]. In a semi-continuous process, pretreatment of textiles significant affected biogas production, due to a more accessible surface area for the degradation of cellulose fibers. Despite the complex structure of cotton/polyester, the initial rate of biogas production was higher and the lag phase shorter in a two-stage batch process then in a single-stage one. Treatment of jeans textiles with N-methylmorpholine-N-oxide (NMMO) and two-stage anaerobic digestion enabled the use of a higher OLR with a shorter HRT.

In addition to the effect chemical pretreatment on anaerobic digestion of cotton textiles, the effect of the substrate-to-inoculum ratio (SIR) has been recognized as affecting the stability of anaerobic digestion of these textiles. Rodriguez-Chiang and Dahl [89] tested the effect of different SIRs on the biogas production potential of wastewater from microcrystalline cellulose production. An SIR of 0.5 provided the fastest methane production rate and a kinetic constant of 0.24 d^−1^, reaching its total yield on day 8 of incubation. In contrast, an SIR of 2.0 led to process inhibition due to accumulation of acids. Juanga-Labayen et al. [90] investigated the effect of SIRs of 0.5, 1.0, 1.5, and 2.0 on mesophilic anaerobic digestion of digested sludge and treated and untreated cotton textile waste. For treatment, 0.5 M Na_2_CO_3_ was added to the cotton textile for 3 h at 150 °C. An SIR of 0.5 and untreated cotton textile waste yielded the largest amount of methane, 366.76 mL/g VS, which is equivalent to an anaerobic degradability of 89.67%. At SIRs of 1.0, 1.5, and 2.0, more methane was produced, and the anaerobic degradability was more than 50% higher with treated than with untreated cotton textile waste. At these three SIRs, treated cotton textile waste yielded similar amounts of methane, approximately 306 mL CH_4_/g VS, equivalent to an anaerobic degradability of 75%.

Cotton textile waste contains more than 52% cellulose; it has a high water retention capacity, a sufficient carbon-to-nitrogen ratio and a low heavy metal content, which all indicate that it can be economically converted into biogas and manure. To convert cotton waste into slurry before loading it into a digester, it requires cleaning and some retention time after mixing with water. Raj et al. [91] investigated biogas production with inoculum (cow dung, pig dung, and goat waste) contents of 2.5%, 5%, 7.5%, 10% and 15%. With 5 kg of cotton waste, about 200 L of biogas containing 77% methane could be generated in 50 days in mesophilic conditions.

Hasanzadeh et al. [92] investigated biogas production using denim jeans, which consisted of a blend of cotton and polyester fibers, as feedstock. They first treated the jeans with calcium carbonate (0.5 and 1 M) at different temperatures (50, 100, 150 °C) to reduce the crystallinity of the cotton, making it easier for the subsequent enzymatic treatment to catalyze hydrolysis. Addition of a glucose solution was tested in anaerobic digestion of the pretreated cotton for biogas production. The maximum methane yields of 328.9 and 361.1 mL/g VS were obtained with pure cotton and jeans, respectively, after pretreatment with 0.5 M Na_2_CO_3_ at 150 °C for 120 min.

Optimizing biogas production requires proper selection of substrates. Kabir et al. [93] explored the performance of AD of wheat straw and wool textile waste alone as well as in co-digestion using wet, semi-dry and dry-AD processes. The wool textile waste consisted of 70% wool (protein) and 30% polyamide, and the wheat straw consisted of 35% cellulose, 22% hemicellulose, 18% insoluble lignin and 16% extractives. A substrate to inoculum ratio of 2:1 was used (based on the volatile solids (VS) content), and the initial total solids (TS) content in the reactor varied from 6% to 30%. The methane production rate was higher with co-digestion of a carbon-rich substrate (wheat straw) and a nitrogen-rich substrate (wool textile waste) than predicted based on the methane potentials of the individual substrates. The degradation of wool textile waste at a TS content of 13% was significantly improved by the addition of a protein-degrading enzyme (0.131 Nm^3^ CH_4_/kg VS). The TS could be increased to 30% during co-digestion without overloading the reactors.

Wool waste could be used as a co-substrate for anaerobic microbial digestion in biogas reactors and thus transformed into renewable energy and liquid fertilizer. Due to its complex structure containing keratin, different pretreatment strategies are needed prior to anaerobic digestion of wool waste. Kabir et al. [94] used two different wool textile wastes to produce biogas and tested different pretreatments (enzymatic with alkaline serine proteinase, thermal at 120 °C for 10 min and a combination of both) for increasing CH_4_ yield. In the combined pretreatment, the samples were first processed thermally and then enzymatically. This combined pretreatment gave the highest methane yield, 0.43 Nm^3^/kg VS, which was 20 times higher than that of controls without any pretreatment.

Liquid nitrogen (LN2) pretreatment improved the solubility and availability to microorganisms of wool waste and did not cause significant changes in its molecular structure. Anaerobic digestion of pretreated wool was up to 80% higher that of untreated wool. It was found that the biogas potential is dependent on the type and source of wool [95].

### 4.2. Composting of Textile Materials

Cotton gin trash, a by-product of the ginning process in textile industry, presents a significant waste disposal problem. The use of uncomposted cotton gin trash as a soil-spread mulch has been extensively studied and demonstrated to cause significant increases in cotton yield when applied at rates from two to six tons per acre. The highest gains were obtained in dryland systems because of the increased water holding capacity, improvement in physical structure and higher content phosphorus and potassium. Composted and vermicomposted cotton trash could act as a good long-term nutrient source, particularly in situations where plant-available potassium and phosphorus levels are limited. The nitrogen content of the final product is increased with composting, although only 7 to 30 per cent of this nitrogen is immediately available. Viable weed seeds and fungal pathogens, including Verticillium wilt, are destroyed during the composting process [96,97].

One possibility for processing waste wool is composting. Composting is more suitable than fertilization with raw wool because it effectively sanitizes the waste, and it is also a great promotor of circular agriculture, due to stabilization of organic waste and production of organic fertilizers [98]. Some examples of waste wool composting have been reported. The effect of composting waste wool in combination with different amounts of cattle slurry as a microbial inoculum and different concentrations of rock phosphate was tested in India [99]. The addition of 10% (*w*/*w*) cattle slurry and 2% (*w*/*w*) rock phosphate resulted in the largest weight loss (27.3%) and the largest decrease in the C:N ratio, from an initial 11.9:1 to 2.06:1, as the result of the degradation of the carbon sources in the compost within 12 weeks. Furthermore, the effect of these composts on yields of chickpea and wheat was tested. Addition of the compost produced from waste wool with 10% (*w*/*w*) cattle slurry and 2% (*w*/*w*) of rock phosphate showed the best results with regard to wheat grain and straw production, as well as chickpea nodulation and grain and straw yield [97]. Composting is also an option for processing low quality or fecal-contaminated wool, especially in the parts of the world where sheep are raised primarily for meat, and has been tested in Texas [100].

### 4.3. Additional Possibilities and Methods Used in Organic Recycling of Textile Waste

In most of the bioprocesses utilizing cotton waste, enzymatic hydrolysis is needed for conversion of cellulose to fermentable sugars [91]. Cellulase consists of multi-enzyme complexes with three functions. Endoglucanase breaks the cellulose polymer chains at random locations along the chain; exo-cellobiohydrolase breaks cellobiose molecules off the cellulose polymer chain from the ends; and beta-glucosidase divides cellobiose into two glucose molecules. Cellulases are the second most-commonly produced industrial enzyme. The majority of commercial cellulase is produced by filamentous fungi via submerged fermentation (SmF) [101]. Unlike solid state fermentation, SmF provides a homogeneous environment and a continuous oxygen supply, as well as better pH control, which can further facilitate cellulase secretion by filamentous fungi. Textile wastes have been used as a substrate for cellulase synthesis in submerged fungal fermentation. The resulting fungal cellulase was subsequently used for textile waste hydrolysis for recovery of glucose and polyester. Using the fungal cellulase, glucose was recovered with a yield of 41.6% [34].

Another process for synthesis of cellulase has been proposed by Hu et al. [102]. Those authors investigated the feasibility of using cotton textile waste as a feedstock to produce cellulase enzymes with the *Aspergillus niger* fungi. Autoclaving was used to facilitate the enzymes’ access to the fibers. The enzymes that were produced were then extracted from the fungi and used to hydrolyze cotton, giving yields of up to 70%.

Textile wastes were utilized as substrates for cellulase production via submerged fungal fermentation. *Trichoderma reesei* ATCC 24449 was selected, and cellulase activity was highest (18.75 FPU/g) after cultivation using a 40/60 cotton/polyester blend as the substrate. The cellulase was used to hydrolyze textile wastes with a glucose recovery yield of 41.6% [103].

A novel biochar-aided approach for processing textile wastes to produce succinic acid has been successfully developed by Li et al. [104]. To hydrolyze mixed textile waste (loading of 9%), cellulase and β-glucosidase were applied at respective dosages of 20 FPU/g substrate and 10 U/g substrate. After enzymatic hydrolysis, a glucose-rich hydrolysate with dyestuff was collected. For removing the colorants, biochar at a dosage of 2 *w*/*w* % was effective. Colorant inhibitors did not inhibit succinic acid synthesis during the subsequent fermentation. To synthesize the succinic acid, *Yarrowia lipolytica* PGC202 and PSA02004 were used. An in situ fibrous bed bioreactor improved the succinic acid titer up to 28.8 g/L, corresponding to a yield of 0.61 g/g without pH control.

Old cotton garments or clothes and used regenerated cellulose fabrics (such as viscose) mainly consist of cellulose polymers, which could serve as an inexpensive polysaccharide resource for bacterial cellulose (BC) production. BC from cotton cloth hydrolysate was obtained at a yield of 10.8 g/L, which was 83% higher than that from a culture grown on a glucose-based medium. The hydrolysate from fabric treated with the ionic liquid (IL) 1-allyl-3-methylimidazolium chloride ([AMIM]Cl) has potential to serve as a high-quality carbon source for BC production by *Gluconacetobacter xylinus* [27]. Woven nylon matrices show great ability to act as supports for immobilizing several enzymes such as laccases or proteases, and other products such as perfumes or medical drugs. Enzyme immobilization onto nylon matrices is a promising system for bioremediation of contaminated soils and wastewater treatment [105].

## 5. Conclusions

A bio-based textile waste management system should promote the development and application of novel recycling techniques, such as further development of biochemical recycling processes. Financing or other incentives could be used to develop strategies for recycling waste streams that are currently only used for thermal recovery operations.

For an efficient bio-based textile waste management strategy, textiles should be collected separately from municipal waste, which means that such schemes need to be established. It is essential to be able to accurately recognize and sort items according to their material content.

Biochemical recycling could be used to valorize bio-based textile waste, thanks to the high selectivity of bio-catalysts, enzymes and biochemical processes. A method of feedstock recycling that deserves special attention is the enzymatic recycling of textiles. For example, cellulase, an enzyme involved in cellulose hydrolysis, can be synthesized by multiple species of fungi and bacteria that use the carbon from textile waste.

The strong interest in bioethanol production has driven the demand for the development of cheap cellulase. This creates a potential opportunity for the textile recycling industry to process cellulose-based textile materials, such as cotton, viscose and Lyocell, using cellulase enzymes that are developed. The glucose solution produced from this enzymatic treatment could be used for producing bioethanol.

Bio-based textile waste can be considered an alternative resource for renewable energy production and has been tested as a raw material for biogas production and as a feedstock for producing nutrient-rich compost.
